# Genetic diversity of porcine group A rotavirus strains in the UK

**DOI:** 10.1016/j.vetmic.2014.06.030

**Published:** 2014-09-17

**Authors:** Rebecca Chandler-Bostock, Laura R. Hancox, Sameena Nawaz, Oliver Watts, Miren Iturriza-Gomara, Kenneth M. Mellits

**Affiliations:** a University of Nottingham, School of Biosciences, Division of Food Science, Sutton Bonington Campus, Loughborough LE12 5RD, UK; b Virus Reference Department, Public Health England, London, NW9 5HT, UK

**Keywords:** Rotavirus, Porcine, Phylogenetic, Zoonosis

## Abstract

Rotavirus is endemic in pig farms where it causes a loss in production. This study is the first to characterise porcine rotavirus circulating in UK pigs. Samples from diarrheic pigs with rotavirus enteritis obtained between 2010 and 2012 were genotyped in order to determine the diversity of group A rotavirus (GARV) in UK pigs. A wide range of rotavirus genotypes were identified in UK pigs: six G types (VP7); G2, G3, G4, G5, G9 and G11 and six P types (VP4); P[6], P[7], P[8], P[13], P[23], and P[32]. With the exception of a single P[8] isolate, there was less than 95% nucleotide identity between sequences from this study and any available rotavirus sequences.

The G9 and P[6] genotypes are capable of infecting both humans and pigs, but showed no species cross-over within the UK as they were shown to be genetically distinct, which suggested zoonotic transmission is rare within the UK. We identified the P[8] genotype in one isolate, this genotype is almost exclusively found in humans. The P[8] was linked to a human Irish rotavirus isolate in the same year. The discovery of human genotype P[8] rotavirus in a UK pig confirms this common human genotype can infect pigs and also highlights the necessity of surveillance of porcine rotavirus genotypes to safeguard human as well as porcine health.

## Highlights

• This is the first study of rotavirus genotypes circulating in UK pigs.
• Rotavirus transmission between pigs and humans is not thought to be common in the UK.
• Human rotavirus genotype P[8] found in a UK pig.
• The uncommon rotavirus genotype P[32] is widespread in UK pig herds.

## 1 Introduction

Rotaviruses have a broad host range that includes mammalian and avian species. In children, group A rotavirus (GARV) is the leading cause of severe gastroenteritis worldwide, and is associated with significant morbidity and mortality, with most children having been exposed by the time they are 5 years old (Tate et al., 2012). In pigs, rotavirus has a significant economic impact through loss in production and is most prevalent in neonatal pigs (<7 days) and piglets at the time of weaning (21–28 days) (Katsuda et al., 2006, Svensmark et al., 1989). Rotavirus can be transmitted zoonotically between pigs and humans. To date there are no reported studies of rotavirus genotypes in symptomatic UK pigs.

Rotaviruses belong to the *Reoviridae* family. They are non-enveloped, double stranded RNA viruses with a segmented genome. The 11 genome segments code for six structural proteins (VP1–4, 6–7) and six non-structural proteins (NSP1–6). There are eight different serogroups of rotavirus (Group A–H), all of which are found in animals or birds (Kindler et al., 2013, Molinari et al., 2014), but only A–C are found in humans (Estes and Cohen, 1989). Pigs are affected by rotavirus serogroups A, B, C, E and H (Molinari et al., 2014, Pedley et al., 1986). The outer capsid of the virus particle is constituted of VP7 (a glycoprotein) and VP4 (a protease sensitive protein), both elicit neutralising antibodies and form the basis of the dual classification of rotaviruses into G and P types, respectively (Estes and Cohen, 1989, Estes and Kapikian, 2007). To date, 27 G-types and 37 P-types of GARV have been identified (Matthijnssens et al., 2011, Trojnar et al., 2013).

Genotype diversity among rotavirus strains is generated by genetic drift, through the accumulation of point mutations, leading to genetic lineages within genotypes and monotypes within serotypes that possess altered epitopes and specific antibody recognition patterns (Coulson and Kirkwood, 1991). In addition, due to the segmented nature of the rotavirus genome, gene reassortment which can take place during co-infection with more than one strain can lead to further rotavirus strain diversity of co-circulating strains. The widespread presence of rotaviruses throughout the animal kingdom constitutes a large reservoir of rotavirus strains, and interspecies transmission combined with reassortment can lead to the emergence of novel or unusual strains that may spread globally. Numerous reports have described interspecies transmission leading to sporadic cases of human disease with rotaviruses from different animal species origin (Ben Hadj Fredj et al., 2013, Doan et al., 2013, Luchs et al., 2012, Mukherjee et al., 2013, Papp et al., 2013). The emergence of epidemiologically important strains such as G9P[8] globally, G10P[11] in India and G8P[4] in Africa, Europe and the USA, in the human population is postulated to have resulted from reassortment with animal strains leading to host adaptation and spread (De Donno et al., 2009, Iturriza-Gomara et al., 2000a, Jere et al., 2011, Leite et al., 2008, Nyaga et al., 2013, Pietsch et al., 2009, Ramani et al., 2009, Than et al., 2013, Weinberg et al., 2012). Worldwide, common porcine rotavirus genotypes are G3, G4, G5, G11 and P[6], P[7], P[13], P[19], P[23], P[26], P[27] (Martella et al., 2010). In Europe, genotypes G1–6, 9–12 and P[6]–P[10], P[13], P[22], P[23], P[27] and P[32] have been identified in pigs (Collins et al., 2010a, Collins et al., 2010b, Midgley et al., 2012).

The aims of this study were to genotype rotavirus in symptomatic UK pigs, to determine the likelihood of zoonotic transmission between pigs and humans within the UK and to compare porcine rotavirus in the UK to genotypes prevalent in Europe and the rest of the world. The findings from the study will not, in themselves, improve biosecurity but will contribute to a better understanding of the potential threat of zoonosis.

## 2 Methods

### 2.1 Sample collection

**Fig. 1:**
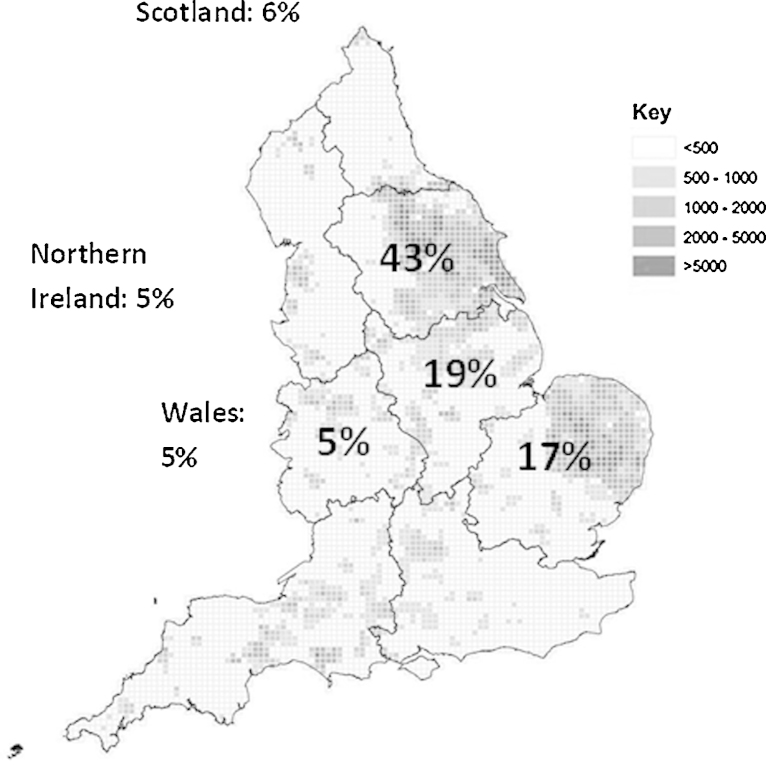
Map of England showing the distribution of pigs per 5 km^2^ in 2010 separated by region, adapted from DEFRA (2010). Percentages on map represent the percentage of samples from each region of England and the percentage taken from Scotland, Wales and Northern Ireland.

Porcine faecal and intestinal content samples were collected from UK pigs; 66% were obtained from the Animal Health Veterinary Laboratories Agency (AHVLA), 34% samples were referred directly to our lab from veterinarians. The samples obtained from the AHVLA had previously tested positive for rotavirus using gel electrophoresis. Other samples were suspected rotavirus infection and were confirmed using RT-PCR (described below). In total, there were 63 samples from 54 different locations between autumn 2010 and spring 2012. The distribution of these samples in the UK is shown in Fig. 1. All samples were obtained and analysed in accordance with the University of Nottingham ethical guidelines.

### 2.2 RNA preparation

Nucleic acid extraction was carried out with QiaXtractor platform (Qiagen) using the specified plastics and the VX reagent kit, as per manufacturers’ instructions, from 10% faecal solutions in Dulbecco's modified Eagle's Medium (DMEM).

### 2.3 RT-PCR amplification of VP7 and VP4

VP7 and VP4 rotavirus genes were amplified from extracted nucleic acids by RT-PCR using methods and primers previously described by Gomara et al. (2001), Gray and Iturriza-Gomara (2011) and Gentsch et al. (1992). Samples producing a band for either VP7 or VP4 were considered positive. Samples that did not amplify in the VP7 and VP4 assays were considered negatives as they were also negative in a VP6-specific qPCR (Gomara et al., 2002). PCR products were purified using QIAQuick PCR Purification Kit (Qiagen) and the forward and reverse strands of VP7 and the VP8* portion of VP4 genes were sequenced using Sanger sequencing (MWG Eurofins) and the same primers as for amplification. Sequences have been added to Genbank database VP7 accession numbers KJ135124–KJ135172 and VP4 accession numbers KJ135173–KJ135220.

### 2.4 Sequence analysis

Genotypes were determined using the RotaC genotyping tool (Maes et al., 2009) and compared to similar sequences using NCBI BLASTn genbank database. Sequence alignments and phylogenetic trees were constructed using Mega6 and ClustalW.

## 3 Results

### 3.1 Rotavirus genotypes in UK pigs

Porcine faecal samples with suspected rotavirus enteritis were obtained from pig producing regions within the UK (Fig. 1). The rotavirus genotypes determined for these samples are shown in Table 1. G4 and G5 were the most common VP7 genotypes, accounting for 25% (16/64) and 36% (23/64) of the strains, respectively. The most common VP4 genotypes were P[6] (33%, 21/64) and P[32] (27%, 17/64). Overall, the most common genotype combinations were G4P[6] and G5P[7].

**Table 1:** Rotavirus genotypes found amongst UK pigs.

	G2	G3	G4	G5	G9	G11	Untyped	Total (%)
P[6]			13	5	2	1		21 (33%)
P[7]			2	9				11 (17%)
P[8]				1				1 (2%)
P[13]		1		1				2 (3%)
P[23]				1				1 (2%)
P[32]		6		4	6		2	17 (27%)
Untyped	2	2	1	2	2	1		10 (16%)

Total (%)	2 (3%)	9 (14%)	16 (25%)	23 (36%)	10 (16%)	2 (3%)	2 (3%)	64

### 3.2 VP4 sequence and phylogenetic analysis

**Fig. 2:**
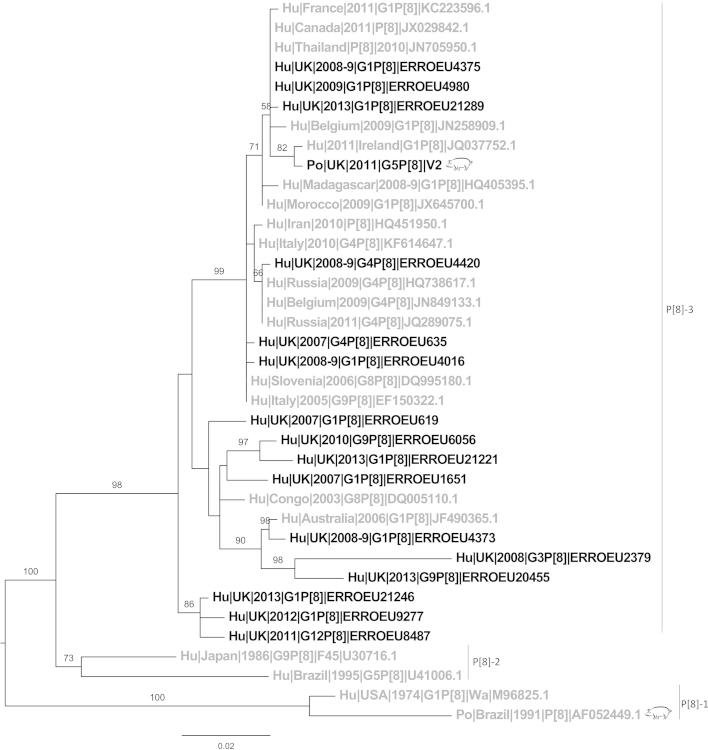
Phylogenetic tree of P[8] sequences. The bars on the right indicate lineages within the P[8] genotype (Iturriza-Gomara et al., 2000a). UK sequences (black), non-UK (grey), porcine sequences (pig symbol), human sequences (no symbol). Bootstrap values more than 50% are shown (1000 pseudoreplicates).

The single P[8] sequence from this study was most similar to a sequence isolated in Ireland in 2011 (Gunn et al., 2012) which shared 99.6% nucleotide identity. This sequence demonstrated high similarity to P[8] rotavirus strains of human origin from the UK and worldwide (93–99%) in lineage P[8]-3 (Fig. 2). The only porcine P[8] sequence available for comparison (Po|Brazil|1991|P[8]|AF052449.1) is Wa-like and found in another lineage (P[8]-1); (Iturriza-Gomara et al., 2000b).

**Fig. 3:**
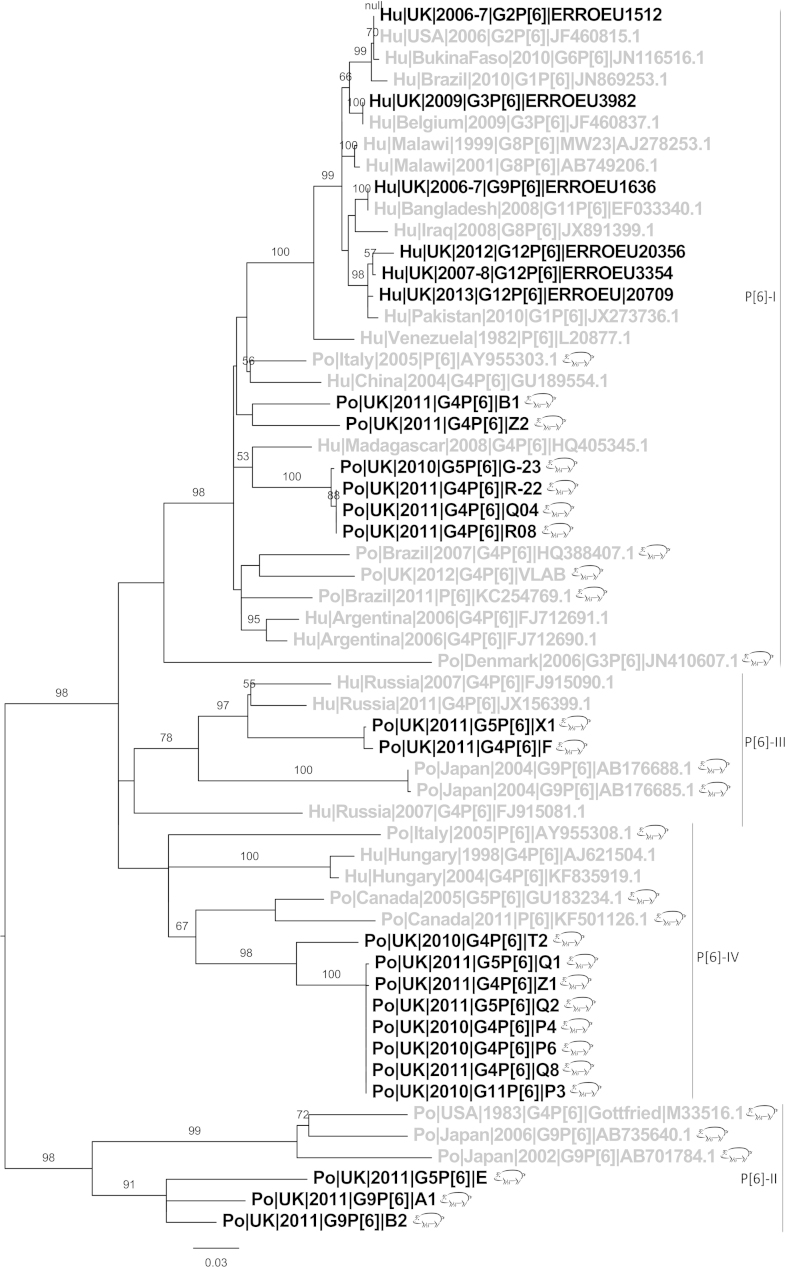
Phylogenetic trees of P[6] sequences. Roman numerals (I, II, III, IV) denote described lineages of P[6] (Martella et al., 2006). UK sequences (black), non-UK (grey), porcine sequences (pig symbol), human sequences (no symbol). UK porcine sequences are all from this study. Bootstrap values more than 50% are shown (1000 pseudoreplicates).

P[6] was the most common (21/64) VP4 genotype (Table 1). Sequences from this study clustered with several P[6] lineages (Fig. 3) (Martella et al., 2006). Three sequences from this study (Po|UK|2011|G9P[6]|A1, Po|UK|2011|G9P[6]|B2 and Po|UK|2011|G5P[6]|E) were most similar to Gottfried strain but shared only 81.4–85.9% identity. Eight sequences from this study were most similar to P[6]-IV lineage but with only 87% identity they are a distinct sub-lineage. The majority of P[6] sequences from this study cluster with P[6]-I lineage sharing 87.1–94.2% identity to most similar reference sequences. There were also two sequences most similar to P[6]-III (84.5–91% identity).

P[32], the next most common genotype (17/54), was found in combination with G3, G5 and G9 (Table 1). The sequences from this study cluster separate to the reference sequences, except for Po|UK|2010|P[32]|E + 41. All sequences from this study except Po|UK|2010|P[32]|E + 41 shared <89% identity with a porcine isolate from the Republic of Ireland and <84% identity with three porcine isolates from Denmark.

### 3.3 VP7 sequence and phylogenetic analysis

**Fig. 4:**
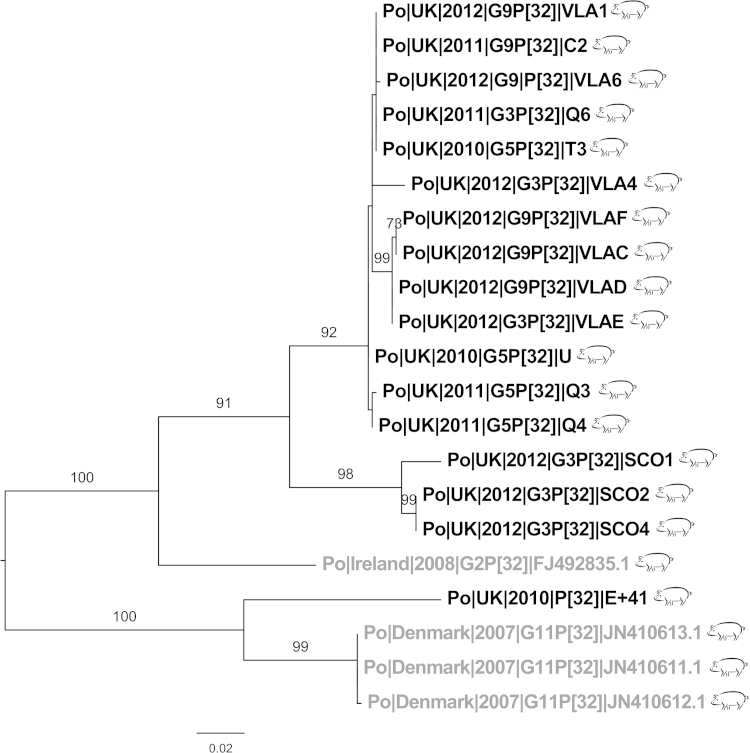
Phylogenetic trees of P[32] sequences. UK sequences (black), non-UK (grey), all sequences are porcine (pig symbol), UK porcine sequences are all from this study. Bootstrap values more than 50% are shown (1000 pseudoreplicates).

**Fig. 5:**
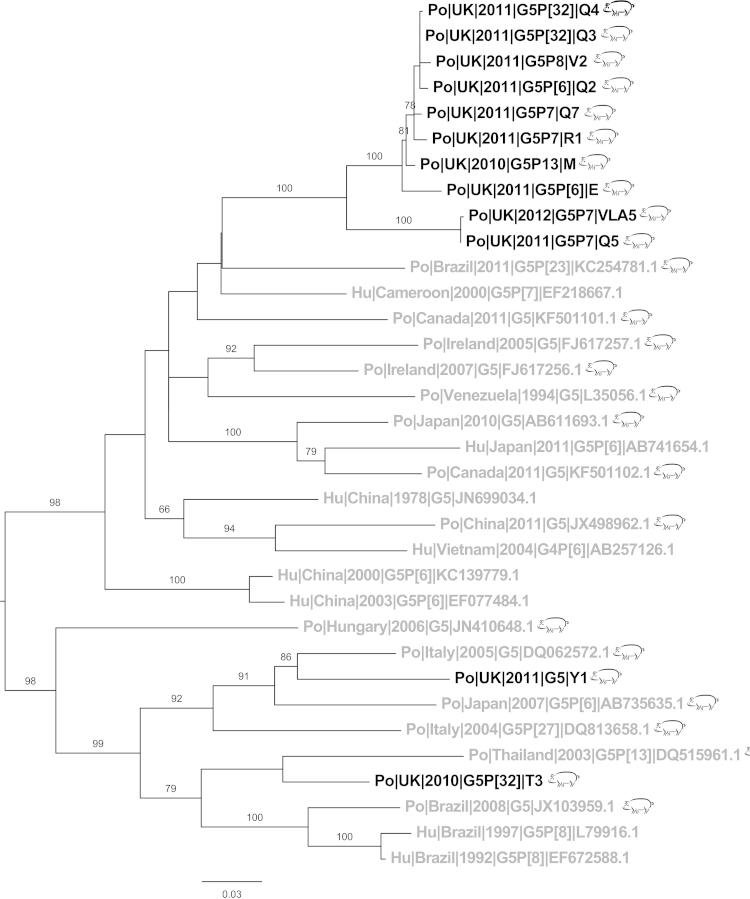
Phylogenetic trees of G5 sequences. UK sequences (black), non-UK (grey), porcine sequences (pig symbol), human sequences (no symbol). UK porcine sequences are all from this study. Bootstrap values more than 50% are shown (1000 pseudoreplicates).

The most common VP7 genotype was G5 (23/62) (Table 1). When analysed, the majority of G5 sequences from this study are clustered, together Fig. 4 sharing a maximum of 89.3% identity to any reference sequence but 92–99.8% identity to each other (Fig. 5). Two of the sequences from this study did not cluster with the rest; Po|UK|2010|G5P[32]|T3 and Po|UK|2011|G5|Y1 were clustered with porcine sequences from Thailand (2005) and Italy (2005, 2006). Irish G5 sequences clustered separately to UK sequences from this study despite being the closest isolates geographically.

**Fig. 6:**
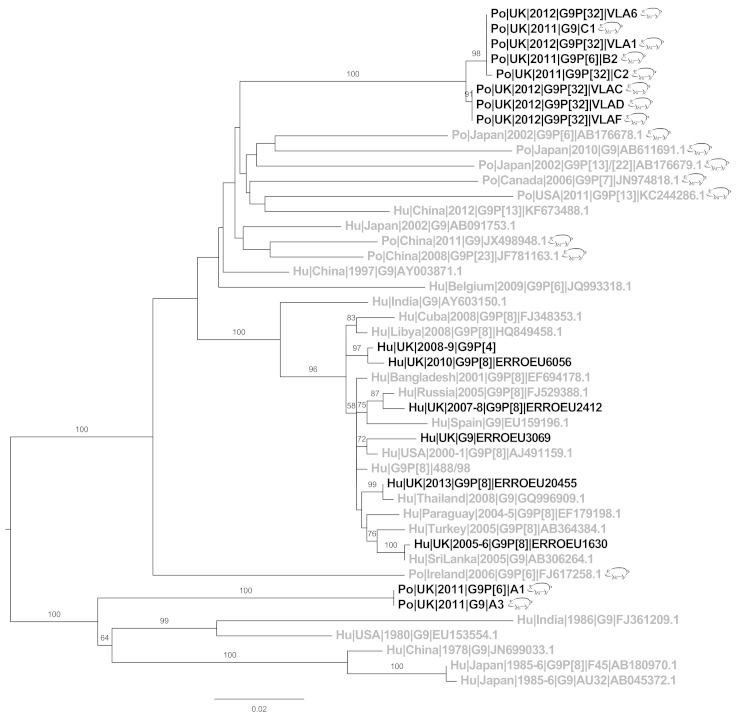
Phylogenetic trees of G9 sequences. UK sequences (black), non-UK (grey), porcine sequences (pig symbol), human sequences (no symbol). UK porcine sequences are all from this study. Bootstrap values more than 50% are shown (1000 pseudoreplicates).

G9 rotavirus sequences (10/62) from this study formed two separate clusters (Fig. 6). The majority of G9 sequences from this study (8/10) clustered together, sharing <94% identity with any reference sequences. The remaining two G9 sequences from this study, Po|UK|2011|G9P[6]|A1 and Po|UK|2011|G9|A3, clustered separately to the other porcine UK sequences, instead with G9 sequences from the 1970s and 1980s isolated in India, China, Japan and USA (89–91% similarity).

## 4 Discussion

### 4.1 High diversity of rotavirus strains in UK pigs

This study describes a wide range of rotavirus genotypes circulating within the UK pig population. Porcine sequences from this study and human rotavirus sequences from the UK were compared to determine the likelihood of zoonotic transmission of rotavirus between pigs and humans in the UK. Although most genotypes found in this study were similar to previous porcine studies, we also identified the P[8] genotype of VP4, which is almost exclusively found in humans, in one isolate (Martella et al., 2010).

The samples from this study represent areas of the UK with the highest density of pig farms (Fig. 1) and hence this survey can be considered representative of rotavirus causing disease in pigs between 2010 and 2012 (DEFRA, 2010). No apparent link between geographical area and genotype was found in the UK. This suggests co-circulating genotypes are spread across the country and may indicate multiple introductions to farms rather than sustained outbreaks.

The vast majority sequences in this study have diverged by >5% from strains circulating in the rest of Europe and the world, for example G5 and G9 (Fig. 5, Fig. 6), likely due to the relative isolation and limited import of pigs to the UK. Indeed the majority of “import” pigs come from the Republic of Ireland (Personal Communication, AHVLA); even so there was still >5% divergence between UK rotavirus and Irish rotavirus sequences at nucleotide level. This observation highlights effective biosecurity between UK pigs and those in neighbouring countries.

### 4.2 Common porcine genotypes in the UK are similar to those worldwide

G4P[6] and G5P[7] were the most common genotype combinations from this study, 20% and 14%, respectively, (Table 1) and they are frequently found in pigs (Martella et al., 2010). G3P[13] and G5P[13], also found in this study, are commonly found in pigs (Chan-It et al., 2008, Miyazaki et al., 2013, Saikruang et al., 2013, Steyer et al., 2008). Four samples from this study had the genotype combination G5P[23], this has only recently been identified in piglets in Brazil (Tonietti et al., 2013), even though both G5 and P[23] are common porcine genotypes (Collins et al., 2010b, Hong Anh et al., 2014, Martella et al., 2010).

### 4.3 Lack of interspecies reassortment of G9 and P[6] in the UK

The G9P[6] genotype has been associated with rotavirus outbreaks in children in the UK and more recently in Belgium (Iturriza-Gomara et al., 2000a, Zeller et al., 2012) and has previously been identified in pigs in the Republic of Ireland and Japan (Collins et al., 2010b, Teodoroff et al., 2005). In this study, porcine and human G9 sequences from the UK did not cluster together, neither did P[6] porcine and human UK sequences (Fig. 3, Fig. 6). Therefore it is unlikely that the zoonotic transmission of G9 and P[6] rotavirus occurred within the UK.

Although the P[6] sequences from this study grouped with representative sequences of P[6] lineages I, II, III and IV (Fig. 3) (Martella et al., 2006), all sequences from this study shared <93% nucleotide identity to any reference sequence. Thus, UK porcine rotaviruses are divergent from European and global sequences. Moreover porcine P[6] sequences from this study did not cluster with the human sequences from the UK, demonstrating a lack of evidence for interspecies reassortment. The multiple lineages of P[6] circulating, suggests multiple introductions of this genotype into the UK pig herd. P[6] is an uncommon human rotavirus genotype in Europe (Cashman et al., 2012, Iturriza-Gomara et al., 2011, Lennon et al., 2008), which may also explain the lack of transmission between the general population and pigs, at least in the UK, who will typically have limited contact with live farm animals.

Worldwide, G9 emerged in humans in the mid 1990s, most likely from pigs as they are the only other species known to be infected by this genotype (Ramachandran et al., 2000). Porcine G9 sequences from the UK form two distinct clusters, both of which are in different lineages to the UK human G9 isolates, which clustered only with other human isolates (Fig. 6) This clear differential clustering of human and porcine rotavirus G9 strains from the UK suggests that interspecies transmission of this genotype was unlikely to have taken place within the UK, and that human and porcine G9 have arrived independently into the UK. The human UK sequences are recorded from 2006, it is impossible to say if there would have been interspecies transmission in this country before that date; however, it is believed that G9 strains only emerged as globally important human rotavirus in the mid 1990s. Prior to that G9 strains had been found in sporadic cases in several countries, and with relative frequency in India, often in association with asymptomatic neonatal infections and with strong evidence of zoonotic transmission (Bhan et al., 1993, Jain et al., 2001, Ramachandran et al., 1996).

### 4.4 The UK contains a rare P[32] genotype

The P[32] genotype was previously identified in Denmark and the Republic of Ireland (Collins et al., 2010a, Midgley et al., 2012). Although these countries are isolated geographically from the UK, pigs do circulate between these countries and the UK (AHVLA, Personal Communication, 2014), and thus is unsurprising that this genotype has been found in the UK as well. Despite having only been recently identified, P[32] is widespread in the UK (found in North Yorkshire, Derbyshire and Scotland). It occurs in combination with G3, G5 and G9, this range of genotypes were likely to be due to reassortment in the UK. The presence of P[32] suggests that UK strains form a distinct pattern different from other European and wider world strains, and therefore it is relevant to survey then as we have here to rationalize any worldwide vaccine.

### 4.5 UK porcine G5P[8] rotavirus is likely to be the result of a reassortment with human P8 and porcine G5 strains

G5P[8] is an uncommon genotype combination. There is only one incidence of G5P[8] isolated from pigs (Gouvea et al., 1999) and few examples of G5P[8] found in humans (Esona et al., 2004, Timenetsky et al., 1997). The main reservoir of the G5 genotype is pigs, but is found sporadically in horses, humans and cattle. P[8] however is the most prevalent human genotype (da Silva et al., 2011, Esona et al., 2004, Martella et al., 2010) it has sporadically been found in pigs and sheep but it was the only genotype found in this study not commonly associated with porcine (Fitzgerald et al., 1995, Gouvea et al., 1999, Halaihel et al., 2010).

The porcine P[8] sequence from this study was most similar to the Irish sequence Hu|2011|Ireland|G1P[8]|JQ037752.1 from an elderly patient in Ireland as part of a study of a rotavirus outbreak in a care home, isolated as G1P[8] (Gunn et al., 2012). This is notable as it was isolated the same year as the P[8] sequence from this study (G5P[8]) was isolated from a pig farm in North Yorkshire. These two sequences share 99.6% nucleotide identity and 99.4% amino acid identity, with only one amino acid difference between the two sequences. This strongly suggests that the two sequences are derived from the same source and that interspecies transmission occurred directly or indirectly to give G5P[8] rotavirus in a neonatal pig. The VP6 genotype of the P[8] isolate from this study was I1, also a human genotype, suggesting the pig was infected with a human rotavirus containing a porcine G5 segment.

All the P[8] sequences from the UK were found in lineage P[8]-3, which is notable considering P[8] is found commonly in the UK (Iturriza-Gomara et al., 2001). The other porcine P[8] (Po|Brazil|1991|P[8]AF052449.1) clustered with P[8]-1 lineage (Iturriza-Gomara et al., 2000b) distinct from the UK P[8] sequence from this study (Fig. 2). P[8] is almost exclusively found in human infections, suggesting adaption of P[8] to the human host,. As with norovirus (NoV) histo-blood group antigens (HBGA's) may determine susceptibility to infection by rotaviruses of different P-types (Huang et al., 2005, Huang et al., 2012). The same HBGA receptors are used for viral attachment of NoV in pigs and humans such as H type 1 receptor (Tian et al., 2007). Huang et al. (2012) postulated that P[8] rotavirus share HBGA receptors with norovirus in the human gut. P[8] and P[4] rotavirus have shown specificity to both Lewis-b and H-type 1 HBGA's. Therefore the lack of P[8] in pigs may be related to pigs having HBGA other than Lewis b. As we have found evidence for zoonotic transmission, a detailed study of the binding capacity of different genotypes to human and animal blood receptors may allow us to better understand constraints to interspecies transmission and predict which strains are more likely to pass between species.

## 5 Conclusion

This study has highlighted a gap in the knowledge regarding UK porcine rotavirus strains, and has found a lack of transmission of porcine rotavirus between the UK and the rest of the world. This information will be useful in the rationalization of genotypes for vaccines to protect UK pigs. An effective vaccine would add significant value to the farming industry (Svensmark et al., 1989), increasing yield of pork, and also may potentially reduce zoonotic transmission. Also, the important discovery of human genotype P[8] rotavirus in a UK pig not only confirms this common human genotype can infect pigs but also highlights the necessity of surveillance of porcine rotavirus genotypes to safeguard human health as well as porcine health.

## References

[bib0005] Ben Hadj Fredj M., Heylen E., Zeller M., Fodha I., Benhamida-Rebai M., Van Ranst M., Matthijnssens J., Trabelsi A. (2013). Feline origin of rotavirus strain, Tunisia, 2008. Emerg. Infect. Dis..

[bib0010] Bhan M.K., Lew J.F., Sazawal S., Das B.K., Gentsch J.R., Glass R.I. (1993). Protection conferred by neonatal rotavirus infection against subsequent rotavirus diarrhea. J. Infect. Dis..

[bib0015] Cashman O., Collins P.J., Lennon G., Cryan B., Martella V., Fanning S., Staines A., O'Shea H. (2012). Molecular characterization of group A rotaviruses detected in children with gastroenteritis in Ireland in 2006–2009. Epidemiol. Infect..

[bib0020] Chan-It W., Khamrin P., Saekhow P., Pantip C., Thongprachum A., Peerakorne S., Ushijima H., Maneekarn N. (2008). Multiple combinations of P 13-like genotype with G3, G4, and G5 in porcine rotaviruses. J. Clin. Microbiol..

[bib0025] Collins P.J., Martella V., Buonavoglia C., O'Shea H. (2010). Identification of a G2-like porcine rotavirus bearing a novel VP4 type, P[32]. Vet. Res..

[bib0030] Collins P.J., Martella V., Sleator R.D., Fanning S., O'Shea H. (2010). Detection and characterisation of group A rotavirus in asymptomatic piglets in southern Ireland. Arch. Virol..

[bib0035] Coulson B.S., Kirkwood C. (1991). Relation of VP7 amino acid sequence to monoclonal antibody neutralization of rotavirus and rotavirus monotype. J. Virol..

[bib0040] da Silva M.F.M., Tort L.F.L., Gomez M.M., Assis R.M.S., Volotao E.D., de Mendonca M.C.L., Bello G., Leite J.P.G. (2011). VP7 gene of human rotavirus A genotype G5: phylogenetic analysis reveals the existence of three different lineages worldwide. J. Med. Virol..

[bib0045] De Donno A., Grassi T., Bagordo F., Idolo A., Cavallaro A., Gabutti G. (2009). Emergence of unusual human rotavirus strains in Salento, Italy, during 2006–2007. BMC Infect. Dis..

[bib0050] DEFRA (2010).

[bib0055] Doan Y.H., Nakagomi T., Aboudy Y., Silberstein I., Behar-Novat E., Nakagomi O., Shulman L.M. (2013). Identification by full-genome analysis of a bovine rotavirus transmitted directly to and causing diarrhea in a human child. J. Clin. Microbiol..

[bib0060] Esona M.D., Armah G.E., Geyer A., Steele A.D. (2004). Detection of an unusual human rotavirus strain with G5P[8] specificity in a Cameroonian child with diarrhea. J. Clin. Microbiol..

[bib0065] Estes M.K., Cohen J. (1989). Rotavirus gene structure and function. Microbiol. Rev..

[bib0070] Estes M.K., Kapikian A.Z. (2007).

[bib0075] Fitzgerald T.A., Munoz M., Wood A.R., Snodgrass D.R. (1995). Serological and genomic characterisation of group A rotaviruses from lambs. Arch. Virol..

[bib0080] Gentsch J.R., Glass R.I., Woods P., Gouvea V., Gorziglia M., Flores J., Das B.K., Bhan M.K. (1992). Identification of group-A rotavirus gene-4 types by polymerase chain-reaction. J. Clin. Microbiol..

[bib0085] Gomara M.I., Cubitt D., Desselberger U., Gray J. (2001). Amino acid substitution within the VP7 protein of G2 rotavirus strains associated with failure to serotype. J. Clin. Microbiol..

[bib0090] Gomara M.I., Wong C., Blome S., Desselberger U., Gray J. (2002). Molecular characterization of VP6 genes of human rotavirus isolates: correlation of genogroups with subgroups and evidence of independent segregation. J. Virol..

[bib0095] Gouvea V., Lima R.C., Linhares R.E., Clark H.F., Nosawa C.M., Santos N. (1999). Identification of two lineages (WA-like and F45-like) within the major rotavirus genotype P[8]. Virus Res..

[bib0100] Gray J., Iturriza-Gomara M. (2011). Rotaviruses. Methods Mol. Biol. (Clifton, NJ).

[bib0105] Gunn L., Feeney S.A., Cashman O., Collins P.J., Coyle P.V., O'Shea H. (2012). Molecular characterization of group A rotavirus found in elderly patients in Ireland; predominance of G1P[8], continued presence of G9P[8], and emergence of G2P[4]. J. Med. Virol..

[bib0110] Halaihel N., Masia R.M., Fernandez-Jimenez M., Ribes J.M., Montava R., De Blas I., Girones O., Alonso J.L., Buesa J. (2010). Enteric calicivirus and rotavirus infections in domestic pigs. Epidemiol. Infect..

[bib0115] Hong Anh P., Carrique-Mas J.J., Van Cuong N., Hoa N.T., Lam Anh N., Duy D.T., Hien V.B., Vu Tra My P., Rabaa M.A., Farrar J., Baker S., Bryant J.E. (2014). The prevalence and genetic diversity of group A rotaviruses on pig farms in the Mekong Delta region of Vietnam. Vet. Microbiol..

[bib0120] Huang P., Farkas T., Zhong W., Tan M., Thornton S., Morrow A.L., Jiang X. (2005). Norovirus and histo-blood group antigens: demonstration of a wide spectrum of strain specificities and classification of two major binding groups among multiple binding patterns. J. Virol..

[bib0125] Huang P., Xia M., Tan M., Zhong W., Wei C., Wang L., Morrow A., Jiang X. (2012). Spike protein VP8* of human rotavirus recognizes histo-blood group antigens in a type-specific manner. J. Virol..

[bib0130] Iturriza-Gomara M., Cubitt D., Steele D., Green J., Brown D., Kang G., Desselberger U., Gray J. (2000). Characterisation of rotavirus G9 strains isolated in the UK between 1995 and 1998. J. Med. Virol..

[bib0135] Iturriza-Gomara M., Dallman T., Banyai K., Bottiger B., Buesa J., Diedrich S., Fiore L., Johansen K., Koopmans M., Korsun N., Koukou D., Kroneman A., Laszlo B., Lappalainen M., Maunula L., Marques A.M., Matthijnssens J., Midgley S., Mladenova Z., Nawaz S., Poljsak-Prijatelj M., Pothier P., Ruggeri F.M., Sanchez-Fauquier A., Steyer A., Sidaraviciute-Ivaskeviciene I., Syriopoulou V., Tran A.N., Usonis V., V.A.N.R M., A.D.E.R Gray J. (2011). Rotavirus genotypes co-circulating in Europe between 2006 and 2009 as determined by EuroRotaNet, a pan-European collaborative strain surveillance network. Epidemiol. Infect..

[bib0140] Iturriza-Gomara M., Green J., Brown D.W., Desselberger U., Gray J.J. (2000). Diversity within the VP4 gene of rotavirus P[8] strains: implications for reverse transcription-PCR genotyping. J. Clin. Microbiol..

[bib0145] Iturriza-Gomara M., Isherwood B., Desselberger U., Gray J. (2001). Reassortment in vivo: driving force for diversity of human rotavirus strains isolated in the United Kingdom between 1995 and 1999. J. Virol..

[bib0150] Jain V., Das B.K., Bhan M.K., Glass R.I., Gentsch J.R. (2001). Great diversity of group A rotavirus strains and high prevalence of mixed rotavirus infections in India. J. Clin. Microbiol..

[bib0155] Jere K.C., Mlera L., O’Neill H.G., Potgieter A.C., Page N.A., Seheri M.L., van Dijk A.A. (2011). Whole genome analyses of African G2, G8, G9, and G12 rotavirus strains using sequence-independent amplification and 454(R) pyrosequencing. J. Med. Virol..

[bib0160] Katsuda K., Kohmoto M., Kawashima K., Tsunemitsu H. (2006). Frequency of enteropathogen detection in suckling and weaned pigs with diarrhea in Japan. J. Vet. Diagn. Invest..

[bib0165] Kindler E., Trojnar E., Heckel G., Otto P.H., Johne R. (2013). Analysis of rotavirus species diversity and evolution including the newly determined full-length genome sequences of rotavirus F and G. Infect. Genet. Evol..

[bib0170] Leite J.P., Carvalho-Costa F.A., Linhares A.C. (2008). Group A rotavirus genotypes and the ongoing Brazilian experience: a review. Mem. Inst. Oswaldo Cruz.

[bib0175] Lennon G., Reidy N., Cryan B., Fanning S., O'Shea H. (2008). Changing profile of rotavirus in Ireland: predominance of P 8 and emergence of P 6 and P 9 in mixed infections. J. Med. Virol..

[bib0180] Luchs A., Cilli A., Morillo S.G., Carmona Rde C., Timenetsky Mdo C. (2012). Rare G3P[3] rotavirus strain detected in Brazil: possible human-canine interspecies transmission. J. Clin. Virol..

[bib0185] Maes P., Matthijnssens J., Rahman M., Van Ranst M. (2009). RotaC: a web-based tool for the complete genome classification of group A rotaviruses. BMC Microbiol..

[bib0190] Martella V., Banyai K., Ciarlet M., Iturriza-Gomara M., Lorusso E., De Grazia S., Arista S., Decaro N., Elia G., Cavalli A., Corrente M., Lavazza A., Baselga R., Buonavoglia C. (2006). Relationships among porcine and human P[6] rotaviruses: evidence that the different human P[6] lineages have originated from multiple interspecies transmission events. Virology.

[bib0195] Martella V., Banyai K., Matthijnssens J., Buonavoglia C., Ciarlet M. (2010). Zoonotic aspects of rotaviruses. Vet. Microbiol..

[bib0200] Matthijnssens J., Ciarlet M., McDonald S.M., Attoui H., Banyai K., Brister J.R., Buesa J., Esona M.D., Estes M.K., Gentsch J.R., Iturriza-Gomara M., Johne R., Kirkwood C.D., Martella V., Mertens P.P.C., Nakagomi O., Parreno V., Rahman M., Ruggeri F.M., Saif L.J., Santos N., Steyer A., Taniguchi K., Patton J.T., Desselberger U., Van Ranst M. (2011). Uniformity of rotavirus strain nomenclature proposed by the Rotavirus Classification Working Group (RCWG). Arch. Virol..

[bib0205] Midgley S.E., Banyai K., Buesa J., Halaihel N., Hjulsager C.K., Jakab F., Kaplon J., Larsen L.E., Monini M., Poljsak-Prijatelj M., Pothier P., Ruggeri F.M., Steyer A., Koopmans M., Bottiger B. (2012). Diversity and zoonotic potential of rotaviruses in swine and cattle across Europe. Vet. Microbiol..

[bib0210] Miyazaki A., Kuga K., Suzuki T., Kohmoto M., Katsuda K., Tsunemitsu H. (2013). Annual changes in predominant genotypes of rotavirus A detected in the faeces of pigs in various developmental stages raised on a conventional farm. Vet. Microbiol..

[bib0215] Molinari B.L., Lorenzetti E., Otonel R.A., Alfieri A.F., Alfieri A.A. (2014). Species H rotavirus detected in piglets with diarrhea, Brazil, 2012. Emerg. Infect. Dis..

[bib0220] Mukherjee A., Mullick S., Deb A.K., Panda S., Chawla-Sarkar M. (2013). First report of human rotavirus G8P[4] gastroenteritis in India: evidence of ruminants-to-human zoonotic transmission. J. Med. Virol..

[bib0225] Nyaga M.M., Jere K.C., Peenze I., Mlera L., van Dijk A.A., Seheri M.L., Mphahlele M.J. (2013). Sequence analysis of the whole genomes of five African human G9 rotavirus strains. Infect. Genet. Evol..

[bib0230] Papp H., Borzak R., Farkas S., Kisfali P., Lengyel G., Molnar P., Melegh B., Matthijnssens J., Jakab F., Martella V., Banyai K. (2013). Zoonotic transmission of reassortant porcine G4P[6] rotaviruses in Hungarian pediatric patients identified sporadically over a 15year period. Infect. Genet. Evol..

[bib0235] Pedley S., Bridger J.C., Chasey D., McCrae M.A. (1986). Definition of two new groups of atypical rotavirus. J. Gen. Virol..

[bib0240] Pietsch C., Petersen L., Patzer L., Liebert U.G. (2009). Molecular characteristics of German G8P[4] rotavirus strain GER1H-09 suggest that a genotyping and subclassification update is required for G8. J. Clin. Microbiol..

[bib0245] Ramachandran M., Das B.K., Vij A., Kumar R., Bhambal S.S., Kesari N., Rawat H., Bahl L., Thakur S., Woods P.A., Glass R.I., Bhan M.K., Gentsch J.R. (1996). Unusual diversity of human rotavirus G and P genotypes in India. J. Clin. Microbiol..

[bib0250] Ramachandran M., Kirkwood C.D., Unicomb L., Cunliffe N.A., Ward R.L., Bhan M.K., Clark H.F., Glass R.I., Gentsch J.R. (2000). Molecular characterization of serotype G9 rotavirus strains from a global collection. Virology.

[bib0255] Ramani S., Iturriza-Gomara M., Jana A.K., Kuruvilla K.A., Gray J.J., Brown D.W., Kang G. (2009). Whole genome characterization of reassortant G10P[11] strain (N155) from a neonate with symptomatic rotavirus infection: identification of genes of human and animal rotavirus origin. J. Clin. Virol..

[bib0260] Saikruang W., Khamrin P., Chaimongkol N., Suantai B., Kongkaew A., Kongkaew S., Ushijima H., Maneekarn N. (2013). Genetic diversity and novel combinations of G4P 19 and G9P 19 porcine rotavirus strains in Thailand. Vet. Microbiol..

[bib0265] Steyer A., Poljsak-Prijatelj M., Barlic-Maganja D., Marin J. (2008). Human, porcine and bovine rotaviruses in Slovenia: evidence of interspecies transmission and genome reassortment. J. Gen. Virol..

[bib0270] Svensmark B., Nielsen K., Dalsgaard K., Willeberg P. (1989). Epidemiological-studies of piglet diarrhea in intensively managed Danish Sow Herds .4. Acta Vet. Scand..

[bib0275] Tate J.E., Burton A.H., Boschi-Pinto C., Steele A.D., Duque J., Parashar U.D. (2012). 2008 estimate of worldwide rotavirus-associated mortality in children younger than 5 years before the introduction of universal rotavirus vaccination programmes: a systematic review and meta-analysis. Lancet Infect. Dis..

[bib0280] Teodoroff T.A., Tsunemitsu H., Okamoto K., Katsuda K., Kohmoto M., Kawashima K., Nakagomi T., Nakagomi O. (2005). Predominance of porcine rotavirus G9 in Japanese piglets with diarrhea: close relationship of their VP7 genes with those of recent human G9 strains. J. Clin. Microbiol..

[bib0285] Than V.T., Kang H., Lim I., Kim W. (2013). Molecular characterization of serotype G9 rotaviruses circulating in South Korea between 2005 and 2010. J. Med. Virol..

[bib0290] Tian P., Jiang X., Zhong W., Jensen H.M., Brandl M., Bates A.H., Engelbrektson A.L., Mandrell R. (2007). Binding of recombinant norovirus like particle to histo-blood group antigen on cells in the lumen of pig duodenum. Res. Vet. Sci..

[bib0295] Timenetsky M., Gouvea V., Santos N., Carmona R.C.C., Hoshino Y. (1997). A novel human rotavirus serotype with dual G5–G11 specificity. J. Gen. Virol..

[bib0300] Tonietti P.O., Hora A.S., Silav F.D., Ruiz V.L., Gregori F. (2013). Phylogenetic Analyses of the VP4 and VP7 Genes of Porcine Group A Rotaviruses in Sao Paulo State, Brazil: rirst identification of G5P[23] in piglets. J. Clin. Microbiol..

[bib0305] Trojnar E., Sachsenroder J., Twardziok S., Reetz J., Otto P.H., Johne R. (2013). Identification of an avian group A rotavirus containing a novel VP4 gene with a close relationship to those of mammalian rotaviruses. J. Gen. Virol..

[bib0310] Weinberg G.A., Payne D.C., Teel E.N., Mijatovic-Rustempasic S., Bowen M.D., Wikswo M., Gentsch J.R., Parashar U.D. (2012). First reports of human rotavirus G8P[4] gastroenteritis in the United States. J. Clin. Microbiol..

[bib0315] Zeller M., Heylen E., De Coster S., Van Ranst M., Matthijnssens J. (2012). Full genome characterization of a porcine-like human G9P[6] rotavirus strain isolated from an infant in Belgium. Infect. Genet. Evol..

