# Corrigendum to “Genetic diversity of porcine group A rotavirus strains in the UK” [Vet. Microbiol. 173 (2014) 27–37]

**DOI:** 10.1016/j.vetmic.2014.10.003

**Published:** 2014-12-05

**Authors:** Rebecca Chandler-Bostock, Laura R. Hancox, Sameena Nawaz, Oliver Watts, Miren Iturriza-Gomara, Kenneth H. Mellits

**Affiliations:** a University of Nottingham, School of Biosciences, Division of Food Science, Sutton Bonington Campus, Loughborough LE12 5RD, UK; b Virus Reference Department, Public Health England, London, NW9 5HT, UK

The authors regret that Kenneth Mellits’ middle initial was listed incorrectly in their paper. The correct initial appears above.

The authors would like to apologise for any inconvenience caused.

